# Description of Stone Morphology and Crystalluria Improve Diagnosis and Care of Kidney Stone Formers

**DOI:** 10.3390/healthcare11010002

**Published:** 2022-12-20

**Authors:** Emmanuel Letavernier, Dominique Bazin, Michel Daudon

**Affiliations:** 1 Institut National de la Santé Et de la Recherche Médicale RM, UMR S 1155, Physiology Unit, Hôpital Tenon, Sorbonne Université, 75020 Paris, France; 2 Laboratoire de Chimie Physique, CNRS UMR 8000, Université Paris Saclay, 91405 Orsay, France; 3 INSERM, UMR S 1155, Physiology Unit, Hôpital Tenon, 75020 Paris, France

**Keywords:** kidney stone, morpho-constitutional analysis, crystalluria

## Abstract

Stone analysis by physical methods is critical to determine their chemical nature and to diagnose the underlying conditions affecting kidney stone formers. This analysis should be completed by a morphologic examination of stone surface and section, leading to the diagnosis of anatomical or metabolic disorders and of specific diseases. Crystalluria study, the analysis of urine crystals, provides complementary information and is extremely useful for both diagnosis and patient follow-up. This review describes briefly how these techniques may be used and in which conditions stone morphology and urine crystal description are particularly relevant for patients medical care.

## 1. Introduction to Stone Morphological Analyses

The analysis of urinary stones is usually performed by physical methods, X-ray diffraction (XRD) or Fourier transform infrared spectroscopy (FTIR), but not by chemical methods that may lead to misdiagnosis [1,2]. The identification of the crystalline components of stones and their relative quantification is necessary to identify the underlying risk factors. Most of stones are made of calcium oxalate (CaOx), calcium phosphate (CaP), uric acid, struvite and cystine, representing together 98% of all urinary stones [2,3,4]. Stones whose main component contains calcium account for more than 80% of total stones. Among them, CaOx monohydrate (COM) or whewellite and CaOx dihydrate (COD) or weddellite are the most frequent crystalline phases, followed by various types of calcium phosphate [1,2,3,5].

Stone analysis is recommended in all kidney stone formers [6]. Physical methods are sometimes sufficient when the stone is made of only one component, such as cystine, 2,8-dihydroxyadenine (DHA) or xanthine, revealing a monogenic disease causative for urolithiasis or when stones are made of struvite and/or ammonium urate, related to chronic urinary tract infection by urea-splitting bacteria. Drugs may also precipitate in urine to form stones. In all these cases, the nature of the stone is closely related to the underlying disease. However, these cases represent a small percentage of all stones, and “pure” stones, i.e., containing only one component, are infrequent [1,2]. By contrast, stones whose main component is CaOx, CaP or uric acid are frequently made of several crystalline phases with a specific spatial organization and specific phenotypes that matter for a more complete diagnosis. 

Actually, if XRD or FTIR analysis is performed on a powdered sample from the whole stone, no information will be obtained regarding the organization and the aspect of the different stone components. For instance, the core of a stone may be made of apatite, coming from a papillary calcification, the stone itself may be made of COM as the main component and the surface of the stone may contain uric acid. The analysis of the powdered sample would reveal mainly COM and, if detected, traces of apatite and uric acid. If performed in parallel, the morphological analysis will tell whether the core of the stone is a Randall’s plaque or a tubular plug, two different origins of kidney stones, and will evidence that uric acid appeared in later stages at the stone surface, potentially revealing the recent development of a metabolic syndrome or diabetes. Morphological examination can also inform whether other stones were present in contact with the stone analyzed, whether the growth of the stone was rapid of if the stone grew up recently [1,2].

In addition, a similar elemental composition, for instance CaOx (COM or COD) or carbonated apatite, may correspond to various medical conditions, and only the specific morphology of both the surface and section of calculi may unravel the underlying disease. Actually, many factors in urine may have an influence on the size of crystals, on their shape, on the kinetics of stone growth and eventually on the stone morphology. 

Michel Daudon’s morpho-constitutional classification was developed in the 1980s as a comprehensive method of stone analysis, combining morphological examination of stone surface and inner structure of the calculus with constitutional analysis by FTIR, allowing identification of the crystalline phases with their relative proportions, aspect and location in the stone [1,2].

## 2. Classification of Kidney Stones According to Stone Composition and Morphology

The method adopted for the morpho-constitutional stone analysis was described extensively [1,2]. To summarize, the protocol encompasses two steps:

First, a morphologic examination of the surface and section of the calculus is performed by using a stereomicroscope. The nucleus and the organization of the inner sections and surface are characterized. Stone size, color, shape, aspect of the surface (for instance smooth, rough, spiky, glazy…) and aspect of the section (organized or not, concentric, radiating…) are recorded. Specific aspects such as the presence of a papillary umbilication and/or Randall’s plaque fragments, and aspect and location of nucleus, are also described.

Second, an analysis is performed by FTIR, from a sample representative of the whole structure (“global powder”) and from parts of the stone more specifically of interest, usually the nucleus, the surface and sometimes the mid-section. 

By using this approach, the stone can be classified into six major types (I–VI), which are subdivided into morphological subtypes corresponding to specific conditions (Table 1, adapted from [1,2]). This classification has been fully described previously. Correlations between medical conditions and the stone morphology and composition were established on the basis of thousands of stones analyzed, for which patients’ medical data and blood and urine biochemistry were also available. 

In the next chapters, the review will focus on specific conditions where stone morphology is particularly relevant for clinicians. 

### 2.1. Calcium Oxalate Stones

CaOx stones may contain COM and/or COD. Stones containing mainly COM are considered as type I stones. Stones containing mainly COD, at least during their formation, are type II stones. Among type I CaOx stones, five different morphologic aspects can be distinguished, referred as type Ia to type Ie. Among type II stones, three different morphologic aspects are described: type IIa, IIb and IIc. The proportion of type I and type II stones is around 20% and 10% in France, respectively [1,2,7]. Mixed type I + II stones (COM + COD) are as frequent as “pure” forms and represent about 20% of all stones. Type I and particularly type II are also frequently mixed with calcium phosphate (type IV).

#### 2.1.1. Type I Stones: COM 

**Figure 1:**
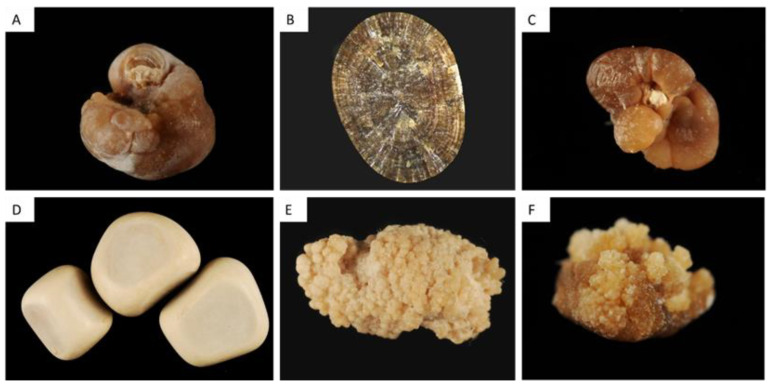
Type I stones, made of calcium oxalate monohydrate. (**A**) Type Ia stone exhibiting a papillary umbilication. The gray veil on stone surface indicates recent crystalline deposits, i.e., stone activity. (**B**) Type Ia stone section showing a concentric and radial organization. (**C**) Type Ia stone with a Randall’s plaque remnant (the whitish deposit in the umbilication). (**D**) Type Id stones, with a smoothened aspect due to stasis and chronic contact with other stones. (**E**)Type Ic stone with a typical whitish and budding structure, due to primary hyperoxaluria. (**F**) type Ie stone, due to enteric hyperoxaluria.

The common determinant of type I stones is an increased CaOx supersaturation in urine with an increased oxalate/calcium ratio [1,2,8]. Figure 1 provides a representative view of the different subtypes of type I stones. Interestingly, FTIR analyses performed on these various types of stones would reveal a similar profile, i.e., typical COM spectra. However, as evidenced by Figure 1, the morphology of type I stones is not uniform, and each morphological subtype corresponds to specific conditions. Urine oxalate levels and circadian modifications of urine oxalate excretion determine the morphological aspect of these subtypes.

##### Type Ia

Type Ia is the most frequent type of “idiopathic” stone, representing 88% of type I stones and 18% of all stones in France [1,2,7]. The surface is dark-brown, smooth and the stone is usually round-shaped. The section is dense with a concentric and radial organization. Risk factors are usually a low diuresis and intermittent hyperoxaluria without hypercalciuria, resulting in a stone growing slowly and incorporating proteins and urine-brown pigments in its structure. A grayish thin superficial deposit may be observed at the stone surface, the significance of which being a recent deposition of COM crystals (Figure 1). Thus, dark-brown Ia stones may be considered as metabolically inactive. By contrast, Ia stones exhibiting a grayish film are discovered in an active stage (Figure 1A).

Type Ia stones frequently exhibit an umbilication, a depression at the stone surface corresponding to the papillary imprint and evidencing thereby the origin of the stone (Figure 1A,C). This umbilication contains frequently CaP deposits corresponding to Randall’s plaque, a calcification process occurring at the tip of the papilla and leading to heterogeneous nucleation of CaOx crystals at its surface and eventually stone formation (Figure 1C) [9,10].

##### Type Ib and Id

In France, Type Ib calculi account for 5% of stones, whereas type Id stones account for 4% [1,2,7].

Type Ib stones are dark-brown and have a mammillary and rough surface with an unorganized section, without umbilication. They correspond to a progressive conversion of COD to COM occurring with time, in patients with intermittent hyperoxaluria and sometimes hypercalciuria and/or urine stasis. 

Type Id stones have a beige or pale brown smooth surface, and a section made of concentric layers without radial organization. These stones correspond to urine stasis (diverticulum or pyeloureteral stenosis) and are smoothened by chronic contact with other stones (Figure 1D). 

##### Type Ic

Type Ic COM stones are infrequent (less than 1% of type I stones) but are pathognomonic for primary hyperoxaluria type I or II (PH1 and PH2), severe medical conditions due to liver genetic defect resulting in massive hyperoxaluria, chronic kidney disease and recurrent stone formation [8,11]. Stones are made of pure COM and exhibit a specific morphology characterized by a pale, yellowish budding surface (without any umbilication) and a loose, unorganized section (Figure 1E). Scanning electron microscopy confirms the distinct crystalline structure of common-type idiopathic COM calculi and of PH1-associated COM stones [12]. 

The identification of a type Ic stone should lead to genetic exams and to specific treatments to decrease oxalate synthesis by liver (pyridoxine, lumasiran, nedosiran, stiripentol) and to decrease urine calcium oxalate supersaturation (increased fluid intakes, potassium citrate…) [11].

##### Type Ie

Type Ie stones represent 2% of type I stones but are also highly suggestive of underlying pathological conditions [1,2,7]. These stones are frequently due to intermittent massive hyperoxaluria due to malabsorption states such as those observed in inflammatory intestinal disease (Crohn’s disease), pancreatic exocrine defects, short bowel syndrome and bariatric surgery associated with malabsorption [13,14]. Type Ie stones exhibit locally budding and poorly organized brown-yellow pale areas mixed with locally concentric dark brown layers with a radiating organization (Figure 1F). This structure is intermediate between type Ia and type Ic and corresponds to periods of fluctuating urine oxalate excretion, probably in relation with diet and intestinal disease activity.

The identification of type Ie stones should lead to specific therapeutic strategies limiting oxalate absorption (specific diet, calcium intakes), and decreasing urine oxalate supersaturation (increased fluid intakes, potassium citrate…).

Another condition associated with type Ie stones is type 2 diabetes (personal data). To date, the underlying mechanisms causative for increased urine oxalate excretion in some patients with type 2 diabetes remains poorly understood but may involve metabolic switches and glyoxal metabolism [15]. 

#### 2.1.2. Type II Stones: COD

**Figure 2:**
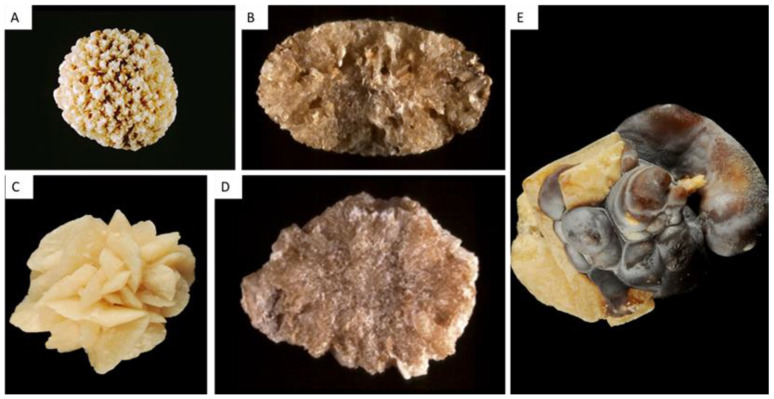
Type II stones, made of calcium oxalate dihydrate (and monohydrate if conversion occurs). (**A**) Type IIa stone with a typical sharp aspect. (**B**) Type IIa stone section, poorly organized. (**C**) Type IIb stone, with less sharp crystallites than type IIa. (**D**) Type IIb stone section. (**E**) Mixed type Ia and IIb stone with crystalline conversion (transformation from weddellite from type II parts into whewellite). Note the presence of Randall’s plaque in the right part of the stone, in the umbilication.

The common determinant of type II stones is an increased CaOx supersaturation in urine with an increased calcium/oxalate ratio (hypercalciuria) [8]. Figure 2 provides representative pictures of type II stones surface and section morphology. 

##### Type IIa

Type IIa stones represent more than two thirds of COD stones in France [1,2,7]. Type IIa stones have a light-brown color and a specific spiculated aspect due to the octahedral and dodecahedral crystallites forming the calculi. The section is radial and poorly organized (Figure 2A,B).

##### Type IIb

Type IIb calculi account for 30% of COD stones [1,2,7]. Their surface is light-brown, surface crystals are eroded and less sharp than type IIa stones, and the section is dense and poorly organized. (Figure 2C,D).

##### Type IIc

Type IIc stones are not frequent and account for less than 1% of COD calculi [1,2,7]. Their surface is pale brown, and the structure is concentric in the periphery of the stone. Type IIc stones are due to the combination of hypercalciuria and stasis of multiple stones in the same environment.

#### 2.1.3. Mixed COM and COD Stones (Type I + II)

These stones are frequent and observed in patients with both hypercalciuria and intermittent hyperoxaluria, most often of dietary origin (Figure 2E). COD crystals may convert to COM with time, as COM is thermodynamically more stable [8]. These stones may form on Randall’s plaques (Figure 2E). Stone morphology is particularly important in this case. Actually, FTIR will reveal a predominance of COM, due to the conversion of COD to COM. The presence of bipyramidal crystals advocates for the role of hypercalciuria in the formation of these stones, which cannot be suspected if the analysis is limited to FTIR. 

#### 2.1.4. Caoxite Stones

Caoxite or calcium oxalate trihydrate (COT) is a rare and unstable crystalline form of calcium oxalate identified in urinary calculi and crystals [1,2]. These rare stones occur in conditions of massive hyperoxaluria due to PH1 or to specific drugs. Stone morphology is very peculiar due to the hexagonal structure of the crystals.

### 2.2. Uric Acid and Urate Stones (Type III)

#### 2.2.1. Type IIIa 

**Figure 3:**
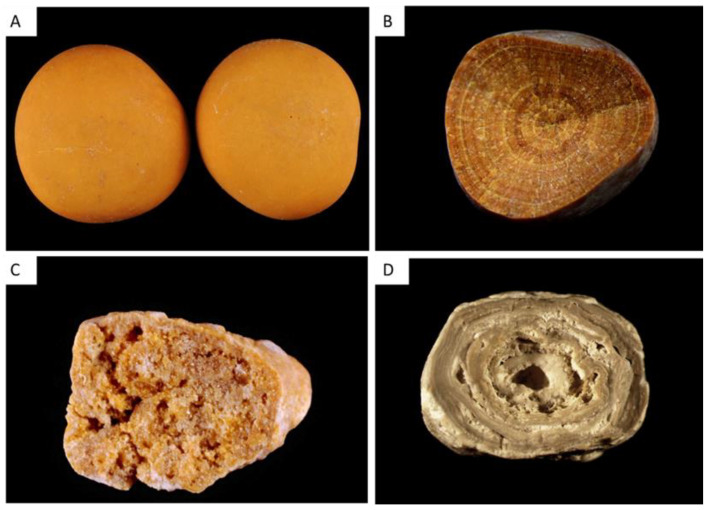
Type III stones, made of uric acid or urates. (**A**) Type IIIa stone with a typical smooth surface and orange color. (**B**) Type IIIa stone section, with concentric layers. (**C**) Type IIIb stone section, with a porous structure. (**D**) Type IIId stone section showing alternatively thick, brown and thin, clear layers with small porous zones.

Type IIIa stones are made of anhydrous uric acid. They have typically a smooth orange surface (but sometimes yellow to ochre) and their section is radial with concentric layers (Figure 3A,B). Uric acid stones are observed in patients affected by low urine pH due to diabetes or metabolic syndrome [4,16,17]. More specifically, type IIIa stones are observed in patients affected by bladder stasis, especially males affected by prostate hypertrophy [1,2,7].

#### 2.2.2. Type IIIb

Type IIIb stones are mainly made of uric acid dihydrate [1,2,7]. Their porous surface has a typical orange color (sometimes whitish or yellow). The section is orange, poorly organized and porous (Figure 3C). Type IIIb stones account for 77% of pure uric acid stones and 7–8% of all stones. They are observed in patients affected by low urine pH (diabetes or metabolic syndrome) and, inconstantly, increased uric acid excretion in urine [4,16]. Uric acid stones type IIIb are also observed in patients with ileostomy resulting in a low-volume urine with a very low pH [18].

Of note, mixed IIIb and Ia type is relatively frequent and observed in patients affected by diabetes or metabolic syndrome with intermittent hyperoxaluria.

#### 2.2.3. Type IIIc

Type IIIc stones are rare in industrialized countries (overall, IIIc and IIId stones represent less than 0.5% of stones) [1,2,7]. Type IIIc calculi are gray or beige with a porous surface, and their section is not organized. These calculi contain urate salts, especially ammonium hydrogen urate, and to a lesser extent sodium urate, and rarely potassium or calcium urate. These stones are formed when urine pH increases, due to urinary tract infection by urease-splitting bacteria or excessive therapeutic urine alkalinization.

#### 2.2.4. Type IIId

Type IIId stones have a rough and porous surface with a dark-brown color and a section showing alternatively thick, brown and thin, clear layers with small porous zones (Figure 3D). They are made of ammonium hydrogen urate. They result from chronic diarrhea of infectious origins in subjects eating an imbalanced diet with low phosphate intakes, and in patients abusing laxative. In developing countries, type IIId calculi are frequently observed in children affected by endemic bladder stones [2,19].

### 2.3. Phosphate Stones (Type IV)

Type IV stones encompass stones made of phosphate, especially CaP (IVa, IVd and partly IVb) and magnesium ammonium phosphate hexahydrate or struvite (IVc and partly IVb). Stones containing CaP as their main component account for 8.6% of stones but CaP is frequently present in other stone types as a minor component. CaP precipitates in urine when pH increases (above 6 and frequently above 6.5) [1,2,7]. Increase in urine calcium and in phosphate concentration also promote the development of these stones if the pH is relatively high.

Carbonated apatite or carbapatite is the most frequent crystalline phase described in CaP stones and is the main component of 67% of these stones. Brushite (dicalcium phosphate dihydrate) is the main component of 14% of stones made of CaP. The other crystalline phases are less frequent and encompass amorphous carbonated calcium phosphate (ACCP), struvite or magnesium ammonium phosphate hexahydrate, octacalcium phosphate pentahydrate (OCP) and whitlockite (a calcium and magnesium phosphate) [1,2,7].

#### 2.3.1. Type IVa1

**Figure 4:**
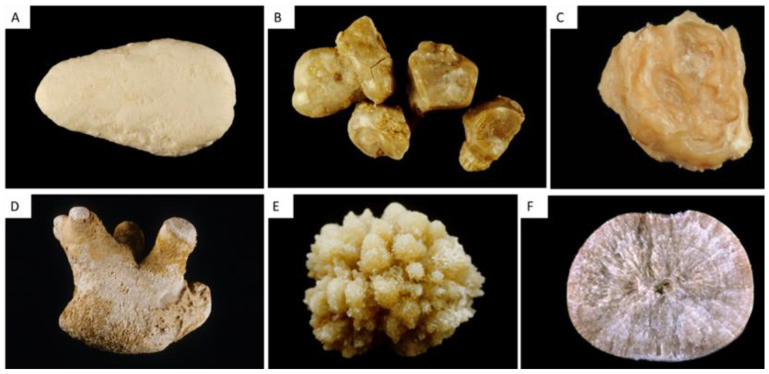
Type IV stones, made of calcium phosphate(s) or struvite. (**A**) Type IVa1 stone with a homogeneous smooth surface. (**B**,**C**) Type IVa2 stone surface and section, with yellow-brown and glazy surface with cracks, and a section showing irregular yellow-brown concentric layers. (**D**) Type IVb complex stone, made of carbapatite admixed with other calcium phosphates or struvite. Type IVb stones have a heterogeneous surface, embossed and rough, and a clear to dark brown color. (**E**,**F**) Type IVd stone surface and section showing slightly rough surface resembling a cabbage, whitish to beige, and a section showing radial crystallization with locally concentric layers.

The most classic CaP stones, known as type IVa1, contain mainly carbapatite, which is often associated with COM or COD in small amounts. These CaP calculi are whitish, their surface is relatively homogeneous and their section contains concentric layers that are poorly organized, with whitish alternate layers (Figure 4A). These stones result from hypercalciuria and/or renal phosphate leak when urine pH is relatively high, and in some cases to urinary tract infection but not necessarily due to urea-splitting bacteria.

#### 2.3.2. The Type IVa2 Carbapatite Subtype and Distal Renal Tubular Acidosis

Some stones made of carbapatite have a very peculiar morphology, with a yellow-brown and glazy surface with cracks, and a section showing irregular yellow-brown concentric layers (Figure 4B,C). Scanning electron microscopy evidences the very different structure of types IVa1 and IVa2 [20].

Type IVa1 and IVa2 are similarly made of carbapatite, and their respective FTIR spectra are quite similar.

However, IVa2 stones correspond to very specific medical conditions and are pathognomonic of a defect in distal tubular acidification. The identification of IVa2 stones may therefore lead to the diagnosis of congenital distal renal tubular acidosis (dRTA), Sjögren’s syndrome, or severe forms of medullary sponge kidney, which induce injury of collecting ducts and localized acidification defects. Only morphological examination of the stone can provide this critical information.

#### 2.3.3. Type IVb

Type IVb calculi contain carbonated apatite but also other calcium phosphates, whitlockite and struvite. Their morphology is typical, with a rough, light to dark brown and heterogeneous surface (Figure 4D). Type IVb section contains whitish and brown alternate layers. These stones reveal the presence of urinary tract infection, even if the proportion of struvite is low [1,2,7]. They usually contain bacterial imprints [21]. 

#### 2.3.4. Type IVc

Type IVc stones are made of struvite and, although their incidence is low (1.2% of stones in our series), they are pathognomonic for severe chronic infection by urea-splitting bacteria [1,2,7]. Struvite calculi have a whitish surface and are made of large rhomboedral crystals forming aggregates. The stone section is poorly organized, with a radial crystallization. 

#### 2.3.5. Type IVd

The component type IVd stone is brushite. These stones account for 1.3% of total stones and 14.4% of CaP stones in France [1,2,7]. Type IVd stones have a whitish and rough cabbage-like surface, and their section shows a typical radial organization (Figure 4E,F). Type IVd stones occur in patients affected by hypercalciuria with a urinary pH around 6.4 and are also frequently associated with renal phosphate leak (genetic defect or primary hyperparathyroidism) [22]. 

These stones should be recognized as their recurrence rate is high, they are resistant to extracorporeal shock-wave lithotripsy, and they may reveal pathological conditions as mentioned above.

#### 2.3.6. Scanning Electron Microscopy in CaP Stones

Scanning electron microscopy in CaP stones, especially those made of apatite, may be useful to identify bacterial imprints, highlighting the bacterial origin of the stone. The number of imprints in stones correlates with their carbonation rate of the apatite, a marker of urine alkalinization by bacteria [21].

#### 2.3.7. Mixed CaOx and CaP Stones

**Figure 5:**
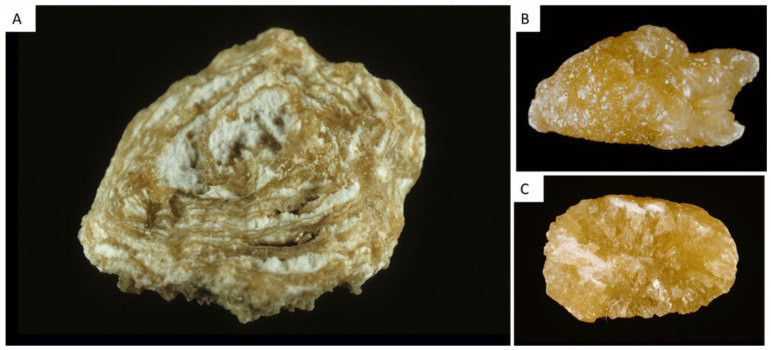
Other classic stones. (**A**) Type IIa + IVa stone with organization in successive concentric layers, characteristic of hypercalciuria associated with hyperphosphaturia. (**B**,**C**) cystine stone surface and section with a yellowish color, a rough surface with hexagonal crystallites and an inorganized section.

The association of CaOx (COM and/or COD) and carbapatite is frequent [1,2,7]. Small amounts of CaP are frequently identified in type II stones and are related to hypercalciuria. Some specific morphologies, and particularly the IIa/b + IVa1 with an organization in successive concentric layers, are characteristic of hypercalciuria associated with hyperphosphaturia, as observed in primary hyperparathyroidism for instance (Figure 5A).

### 2.4. Other Stones

#### 2.4.1. Type V Stones

Type V stones are made of cystine and are due to cystinuria, which can be easily diagnosed by physical methods. Nevertheless, morphological examination provides useful information. Va stones have a typical aspect with a yellowish color, a rough surface with hexagonal crystallites and an inorganized section (Figure 5 B,C). Vb type has the same origin, but the surface is smooth and has a creamy color, with concentric layers at the periphery and an unorganized core. Type Vb may be due to an anatomical confinement. Some CaP deposits may be observed when therapeutic alkalinization is not associated with a sufficient diuresis.

#### 2.4.2. Type VI Stones

Type VIa stones are soft light-brown matrices due to urinary tract infections.

Type VIb stones are made of concentric protein layers covered by crystals from metabolic infectious or drugs, usually brown, but various colors may be observed.

Type VIc stones are small and made of unorganized agglomerates of COM and proteins with a dark brown shell of proteins. These stones are observed in patients affected by end-stage renal disease, with very low urine excretion, but persistent oxaluria and proteinuria.

#### 2.4.3. Other Types

Many drugs may result in stone formation, when drugs or metabolites precipitate in urine, sometimes with specific aspect. Monogenic disorders such as APRT enzymatic deficiency or xanthinuria lead to the formation of specific stones, with different morphologies that were extensively described [1,2,23].

## 3. Morpho(-Constitutional) Analysis of Kidney Stones: Clinical Relevance

As described above, the description of stone morphology provides original clinical information and may distinguish the different crystalline phases according to the period of stone formation. The core of a stone may differ from section or surface, highlighting the succession of different risk factors with time.

The observation of crystalline conversion from COD to COM reveals that a stone made mainly of COM was initially due to hypercalciuria and COD.

The presence of Randall’s plaque remnants in stone umbilication reveals their origin and the presence of massive Randall’s plaque in renal papilla, with a potential risk for stone recurrence.

Type Ic and Ie reveal the presence of massive hyperoxaluria, due to PH or enteric hyperoxaluria, respectively.

Anatomical confinement may be detected when Id, IIc or IIIa stones are observed.

The IVa2 type is pathognomonic of distal tubular acidosis and may reveal the presence of a genetic disease or of Sjögren syndrome.

In addition, the organization between crystalline phases or the presence of various type of stones in an individual also provide useful information. Actually, type II + IV stones reveal the presence of resorptive hypercalciuria, and potentially primary hyperparathyroidism. In patients affected by medullary sponge kidney, stones may be pleiomorphic: the association of type I, type II, type IVa1 and sometimes IVa2 stones in an individual advocates for the presence of MSK [24].

Therefore, morphological analysis provides essential information that is complementary with data obtained by FTIR and XRD. These analyses should be coupled to metabolic investigations including plasmaand urine biochemistry. In addition to these investigations, crystalluria study is also very useful.

## 4. Introduction to Crystalluria

As stated above, the morphological examination of kidney stones provides rapid, costless and precious clinical information, but the morphological analysis of the crystals identified in urine may also be extremely useful for clinicians [25,26].

Actually, the study of crystalluria:

(i) leads to the diagnosis of severe monogenic diseases (cystinuria, primary hyperoxaluria, adenine phosphoribosyltransferase deficiency)
(ii) identifies crystals due to drugs, which may be responsible for acute or chronic kidney injury
(iii) allows the assessment of metabolic disorders associated with stone formation
(iv) may predict the risk of stone recurrence.

Crystals may form in urine in supersaturation states, i.e., when there is a disequilibrium between crystallization promoters and inhibitors. In human urine, calcium, oxalate, urate and phosphate ions are the most frequent promoters of crystal formation. These promoters may be quantified by urine chemistry, but less frequent promoters, especially drugs and promoters due to genetic diseases (cystine, xanthine, 2,8-DHA), are not quantified routinely. Inhibitors are substances filtered or secreted in urine, decreasing crystal formation, growth, aggregation or adhesion. Among these inhibitors, only citrate and magnesium, and to a lesser extent pyrophosphate, may be assessed by biochemistry. Many macromolecules such as osteopontin, Tamm-Horsfall protein, fetuin-A, matrixGLA protein and others are not quantified in urine but play essential roles [27]. Therefore, the study of crystalluria provides an integrated view of the mechanisms leading to crystal formation, which cannot be assessed by urine biochemistry alone. 

Crystalluria should be investigated by a specific methodology that has been described extensively [25,26]. Briefly, the first morning or the second urine is brought to the laboratory rapidly (<2 h after urination), the urine is stored at room temperature and not exposed to cold temperatures, and crystalluria study should be processed without delay. Crystals are analyzed by using a phase contrast microscope, with a polarized light device, mandatory to study crystals refringence. Urine pH is measured as most crystalline species are pH-sensitive (exceptions: COM, COD, COT and 2,8-dihydroxyadenine crystals). The urine is gently shaken to homogenize the crystals. the microscopic examination encompasses urine cytology, identification of crystals and, depending on crystal type, quantification of crystals and aggregates. For crystals that cannot be identified by crystalluria study, infrared spectroscopy is recommended [26].

## 5. Classification of Urinary Crystals

### 5.1. Calcium Oxalate Monohydrate and Dihydrate: COM and COD

#### 5.1.1. COM or Whewellite

**Figure 6:**
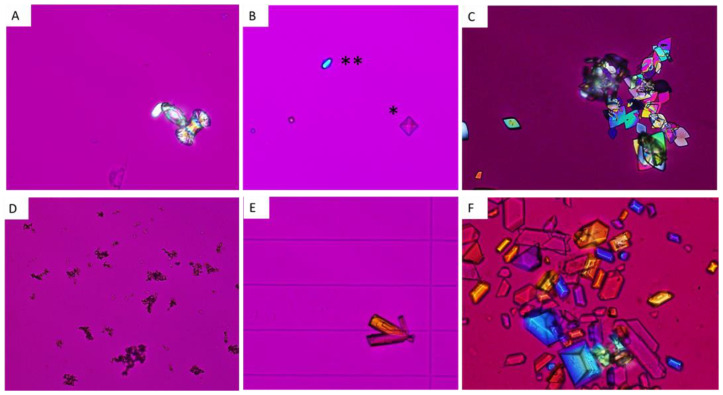
Crystalluria: “metabolic” urine crystals. (**A**) Calcium oxalate monohydrate crystals with a dumb bell aspect. (**B**) Calcium oxalate dihydrate crystals, with both frequent octahedral (*) and dodecahedral shapes (**), which are typical features of hypercalciuria. (**C**) Uric acid dihydrate crystals in an acidic urine. (**D**) Amorphous or poorly crystallized calcium phosphates in a relatively “alkaline” urine. (**E**) Brushite crystals, typical features of hypercalciuria associated with increased urine phosphate excretion. (**F**) Struvite crystals in an alkaline urine, due to urea-splitting bacteria.

COM crystals are associated with high oxalate urine concentration in the presence of normal or low calcium urine concentration [8]. The presence of COM crystals without COD or of a large number of COM crystals is highly suggestive of a very high urine oxalate excretion that may unravel PH1-3 or enteric hyperoxaluria [28]. Some COM crystallites have a typical dumb-bell-like shape due to increased levels of urine oxalate. Smaller COM crystals looking like red blood cells are associated with moderately increased urine oxalate concentration (Figure 6A). After intoxication by ethylene glycol, which is transformed into oxalate by the liver, COM crystals with a specific shape of shuttles or elongated hexagons are observed in urine.

#### 5.1.2. COD or Weddellite

COD appears usually as octahedral (bipyramidal) crystals (Figure 6B). Their presence may reveal hypercalciuria but COD may also form rapidly in urine ex vivo, and COD precipitation may in some cases be due only to a delayed processing of urine sample. By contrast, dodecahedral COD crystals are pathognomonic for hypercalciuria (Figure 6B) [25,26]. Hypocitraturia and hyperoxaluria may increase the size of COD crystals in urine.

### 5.2. Uric Acid and Urate Salts

#### 5.2.1. Uric acid

Uric acid crystals form in acidic urine, especially when urine pH is ≤5.5. Four types of uric acid crystals may be identified: dihydrate uric acid, monohydrate uric acid, anhydrous uric acid and amorphous uric acid [25,26]. Dihydrate uric acid is common and presents usually as flat diamonds, but it may have many different presentations (Figure 6C). It is usually identified when urine pH is very low. By contrast, amorphous uric acid depends on high urate concentration. The two other phases are less frequent.

#### 5.2.2. Urate salts

Urate salts, and most frequently ammonium urate, form in alkaline urine and may be due to urine alkalinization by urea-splitting bacteria (especially if mixed with struvite or CaP crystals) or to increased urate concentration with intermediate to high pH.

### 5.3. CaP Crystals and Struvite

#### 5.3.1. CaP

CaP crystals in urine encompass mainly amorphous carbonated calcium phosphate (ACCP), carbonated apatite, whitlockite (mixed calcium and magnesium phosphate), OCP and brushite [25,26]. The first species cannot be distinguished by crystalluria study and presents as granulations that do not polarize (Figure 6D). They occur when calcium phosphate supersaturation is increased, especially when urine pH is relatively high, above 6.5. By contrast, brushite crystals are typically rod-shaped and may form aggregates (Figure 6E). The formation of brushite requires an increased calcium phosphate supersaturation depending on high urine calcium and phosphate concentration rather than increased urine pH, which is usually intermediate (6–6.5). The presence of brushite may therefore reveal the presence of hypercalciuria, especially if associated with COD and/or renal phosphate leak.

#### 5.3.2. Struvite

Magnesium ammonium phosphate hexahydrate or struvite is pathognomonic for the presence of a urea-splitting bacteria in human urine. The pH is usually high above 7, and crystals have typical rhomboedric coffin shapes and trapezoidal aspects (Figure 6F).

### 5.4. Crystals Due to Monogenic Diseases

Crystalluria is sometimes the best tool to rapidly diagnose gene disorders causative for kidney stone formation but also chronic kidney disease.

#### 5.4.1. Cystine

**Figure 7:**
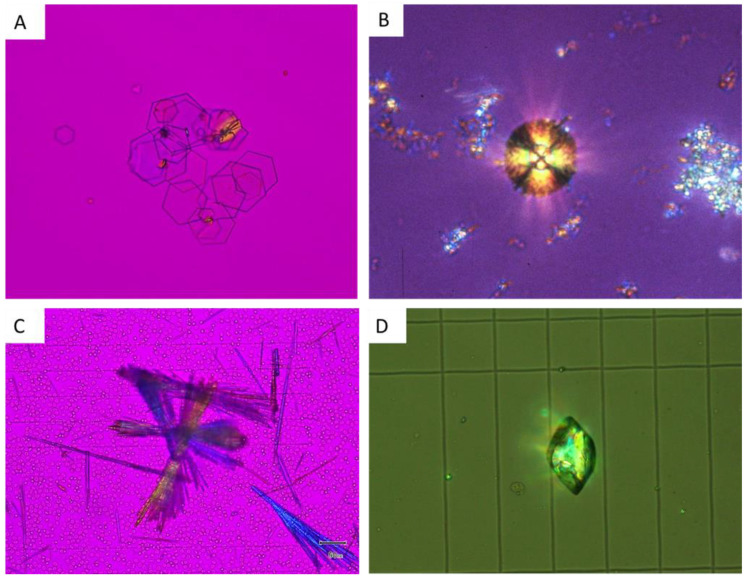
Crystalluria: “genetic” and drug-induced urine crystals. (**A**) Cystine crystals, with a typical hexagonal aspect. (**B**) 2,8 dihydroxyadenine crystals, with the typical “maltese cross” aspect in polarized light. (**C**) amoxicillin crystals in the urine of a patient affected by acute kidney injury. (**D**) sulfamethoxazole crystals in the urine of a patient affected by acute kidney injury.

Cystine crystals are pathognomonic for cystinuria, a monogenic disorder characterized by increased cystine excretion in urine, which is poorly soluble and causative for recurrent stone formation and renal failure in some cases. Crystals are flat, hexagonal, isolated or form aggregates (Figure 7A). The shape of crystals is so typical and their presence so specific that diagnosis is almost certain, even when only a few crystals are observed in urine.

#### 5.4.2. 2,8-Dihydroxyadenine

The genetic invalidation of adenine phosphoribosyltransferase (APRT) enzyme activity results in an increased excretion of 2,8-dihydroxyadenine, which forms crystals in urine due to its poor solubility. Crystallites are spherical, and a typical “maltese cross” appears in the crystals under polarized light (Figure 7B). The diagnosis of APRT deficiency is difficult, as crystals may induce silent chronic kidney disease, leading to end-stage renal failure. The diagnosis of APRT deficiency is, unfortunately, sometimes diagnosed when the disease affects a kidney graft, when crystals are observed in a biopsy [23]. FTIR may be of help to confirm diagnosis on urine crystals as on kidney biopsies.

#### 5.4.3. Xanthine

Xanthine crystals are due to xanthinuria, a genetic disorder due to xanthine oxidase deficiency. Crystals present as unspecific polarizing granules or sticks without a characteristic aspect.

#### 5.4.4. COM

As stated above, a high number of COM crystals reveals hyperoxaluria, potentially related to PH [28].

#### 5.4.5. Others

Tyrosine, leucine or potassium orotate crystals may also reveal underlying genetic disorders but are much less frequent.

### 5.5. Drug-Induced Crystals

Many drugs or their metabolites may precipitate in urine, leading to stone formation but also to acute renal injury. Crystalluria is particularly useful in contexts of acute renal failure in intensive care units or when a specific drug is supposed to precipitate in renal tubules. Crystalluria study evidences typical crystals. FTIR analyses are frequently useful to confirm the nature of crystals [25,26]. Many drug-induced crystals have been characterized.

For instance, amoxicillin crystals precipitate frequently in urine when high amounts of amoxicillin are prescribed to patients affected by meningitis, endocarditis or osteitis. Crystals form aggregates of needles with a brush aspect (Figure 7C) [29,30]. Sulfamethoxazole crystals are also frequently observed and may present similar features with dihydrate uric acid crystals, but their polarization profile is more homogeneous and they may also be extremely pleiomorphic (Figure 7D) [31]. Many other antibacterial and antiviral agents may form crystals in urine, including sulfadiazine and its metabolite N-acetylsulfadiazine, fluoroquinolones, ceftriaxone, foscarnet, acyclovir, atazanavir, etc.

Some toxic compounds, such as high caffeine intake, may also result in 1-methyl uric acid crystal formation, leading to stone formation or crystalline nephropathy [32].

Finally, vancomycin is a drug that may form amorphous deposits harmful for renal tubules, and vancomycin casts may be detected in urine by crystalluria, sometimes with the help of complementary immunostainings [33].

The imputability of crystals in acute kidney injury raises question, as their identification in acute settings does not prove that crystals are causative for acute kidney injury. Nevertheless, the rapid recovery of renal function after drug withdrawal suggests that the drug was actually toxic for renal tubules. Moreover, recent studies dedicated to amoxicillin crystalluria evidenced that the presence of crystals predicts prospectively the onset of acute renal failure [29,30].

## 6. Crystalluria Study Reveals Lithogenic Activity and Predicts Stone Recurrence

Beside its interest for diagnosis, crystalluria study is also indicative for individual lithogenic activity and risk of recurrence [34]. In stone formers, crystalluria is significantly more frequently positive than in healthy subjects, highlighting the harmful role of crystals in stone formation.

Crystalluria analysis was performed in 204 CaOx kidney stones formers at each visit over 7 years. The detection of crystals in ≥50% of examined urine samples was associated with stone recurrence in 87% of patients. By contrast, stone recurrence was observed in only 9% of patients with a less frequent detection of crystals [34]. A ratio or ‘crystalluria index’ was therefore defined as follows: (number of positive urine samples/number of examined samples) in an individual. A crystalluria index > 0.50 indicates that the recurrence rate is dramatically increased.

The absence of crystals in urine indicates that lithogenic activity is low. The amount of crystals is also significant, especially in genetic disorders with a high recurrence rate [23,28,35]. In two conditions, PH1 and cystinuria, the global crystalline volume (GCV) in urine was predictive for recurrence.

In cystinuria, a cystine GCV > 3000 μm^3^/mm^3^ of urine is associated with recurrence [35].

In PH1, a GCV < 500 μm^3^/mm^3^ of urine was predictive for a good prognosis in patients with recent liver–kidney transplantation, a critical period due to urine release of oxalate in urine, previously accumulated in the body [28].

## 7. Conclusions

Both morphological characterization of kidney stones and crystalluria are valuable tools for the diagnosis of urolithiasis, providing information that other techniques cannot obtain. These techniques are cheap but time consuming and require a specific training. However, the improvement of diagnosis, especially in severe inherited disorders, supports the extensive use of this approach. Moreover, patient follow-up may be improved by using crystalluria systematically to detect patients at risk for recurrence and correct the underlying metabolic disorders.

## Figures and Tables

**Table 1:** Morpho-constitutional classification of urinary calculi, adapted from [1,2].

Type	Main Component	Surface	Section
Ia	Whewellite	Mammillary surface. Frequent umbilication and Randall’s plaque indicative of papillary origin. Color: brown.	Section made of concentric and compact layers with a radiating organization. Color: brown.
Ib	Whewellite	Mammillary and rough surface. No umbilication. Color: brown to dark brown.	Compact unorganized section. Sometimes, the presence of gaps. Color: brown to dark brown.
Ic	Whewellite	Budding surface. Light color, cream to pale yellow-brown, sometimes whitish in children.	Finely granular and poorly organized section. Light color, cream to pale yellow-brown.
Id	Whewellite	Smooth surface. Color: homogeneous, beige or pale brown.	Compact section made of thin concentric layers. Color: pale brown or beige.
Ie	Whewellite	Locally budding, mammillary or rough surface. Color: often heterogeneous, pale yellow-brown to brown.	Locally unorganized and loose structure, locally more compact radiating structure. Color: heterogeneous, pale yellow-brown to brown.
IIa	Weddellite	Spiculated surface showing aggregated bipyramidal crystals with right angles and sharp edges. Color: pale yellow-brown.	Section showing loose radial crystallization. Color: pale yellow-brown.
IIb	Weddellite	Spiculated surface showing aggregated bipyramidal crystals with blunt angles and ridges. Color: pale yellow-brown.	Section showing compact unorganized crystallization. Color: pale yellow-brown.
IIc	Weddellite	Rough surface. Color: gray-beige to dark yellow-brown.	Unorganized core with a diffuse concentric compact structure at the periphery. Color: gray-beige to dark yellow-brown.
IIIa	Uric acid anhydrous	Homogeneous smooth surface. Color: homogeneous, typically orange, sometimes cream, ochre or yellowish.	Homogeneous compact, concentric structure with a radiating organization. Color: typically orange.
IIIb	Uric acid dihydrate ± uric acid anhydrous	Heterogeneous embossed, rough and porous surface. Heterogeneous color from beige to brown-orange.	Poorly organized section with frequent porous areas. Color: orange.
IIIc	Urate salts, including ammonium hydrogen urate	Homogeneous or slightly heterogeneous rough and locally porous surface. Color: homogeneous, cream to grayish.	Unorganized porous section. Color: whitish to grayish.
IIId	Ammonium hydrogen urate	Heterogeneous embossed, rough and porous surface. Heterogeneous color: grayish to dark brown.	Section made of alternated layers, thick and brownish or thin and whitish to grayish, locally porous. Sometimes, locally purplish.
IVa1	Carbapatite	Homogeneous rough surface. Color: whitish to beige.	Section: poorly organized, or diffuse concentric layers. Color: whitish to beige.
IVa2	Carbapatite	Embossed and varnished surface with small cracks. Glazed appearance. Color: homogeneous, pale brown-yellow to pale brown.	Section made of compact alternated layers, thick brown-yellow and thin beige. Often, multiple nuclei (from collecting duct origin).
IVb	Carbapatite + other calcium phosphates (±struvite)	Heterogeneous, both embossed and rough surface with confluent superficial deposits. Heterogeneous color: cream to dark brown.	Section made of irregularly alternating thick, whitish, and thin, brown-yellow layers.
IVc	Struvite	Homogenous surface made of amalgamate crystals with blunt angles and edges.	Section: crude radial crystallization. Color: whitish.
IVd	Brushite	Finely rough or dappled surface. Color: whitish to beige.	Radial crystallization with more or less visible concentric layers. Color: whitish to beige.
Va	Cystine	Rough surface. Color: yellowish.	Section: poorly organized, sometimes a radiating organization. Color: yellowish.
Vb	Cystine	Smooth surface. Color: homogeneous, cream to yellowish.	Concentric layers at the periphery, an unorganized core. Color: heterogeneous, cream (periphery) to yellowish (core).
VIa	Proteins	Matrix soft calculi. Homogeneous surface. Color: cream to pale brown.	Unorganized section. Color: cream to pale brown.
VIb	Proteins ± drugs or metabolic compounds	Heterogeneous, irregularly rough surface. Locally scaled. Color: dark brown to black.	Crude and diffuse foliated structure. Color: dark brown to black. Other components often present in these stones may alter the structure and the color.
VIc	Proteins + whewellite	Homogeneous, smooth surface with clefts and scales. Color: dark brown.	Section made of a dark brown protein shield surrounding a loose, unorganized light core containing whewellite crystals mixed with proteins.
VII	Miscellaneous	Various morphologies and colors according to the stone composition (infrequent purines and drugs).	Variable organization and color according to the stone composition.
